# T Cell Migration in Three-dimensional Extracellular Matrix: Guidance by Polarity and Sensations

**DOI:** 10.1155/2000/56473

**Published:** 2000

**Authors:** Peter Friedl, Eva-Bettina Bröker

**Affiliations:** Cell Migration Laboratory Department of Dermatology University of Würzburg Josef-Schneider-Str. 2. 10 Würzburg 97080 Germany

**Keywords:** T cell locomotion, three-dimensional extracellular matrix, integrins, contact guidance

## Abstract

The locomotion of T lymphocytes within 3-D extracellular matrix (ECM) is a highly dynamic and flexible process following the principles of ameboid movement. Ameboid motility is characterized by a polarized yet simple cell shape allowing high speed, rapid directional oscillations, and low affinity interactions to the substrate that are coupled to a low degree of cytoskeletal organization lacking discrete focal contacts. At the onset of T cell migration, a default program, here described as migration-associated polarization, is initiated, resulting in the polar redistribution of cell surface receptors and cytoskeletal elements. Polarization involves protein cycling either to the leading edge (i.e. LFA-1, CD45RO, chemokine receptors, focal adhesion kinase), to a central polarizing compartment (MTOC, PKC, MARCKS), or into the uropod (CD44, CD43, ICAM- and –3, β1 integrins). The function of such compartment formation may be important in chemotactic response, scanning of encountered cells, and a flexible and adaptive interaction with the ECM itself. Due to the simple shape and a diffusely organized cytoskeleton, the interactions to the surrounding extracellular matrix are rapid and reversible and appear to allow a broad spectrum of molecular migration strategies. These range from (1) adhesive and haptokinetic following i.e. chemokine-induced motility across 2-D surfaces to (2) largely integrin-independent migration predominantly guided by shape change and morphological flexibility, as seen in 3-D type I collagen matrices. Their prominent capacity to rapidly adapt to a given structural environment coupled to contact guidance mechanisms set T cell locomotion apart from slow, focal contact-dependent and more adhesive migration strategies established by fibroblast-like cells and cell clusters. It is therefore likely that, within the tissues, besides chemotactic or haptotactic gradients, the preformed matrix structure has an important impact on T cell trafficking and positioning in health and disease.


*Developmental Immunology*, 2000, Vol. 7(2-4), pp. 249-266
Reprints available directly from the publisher
Photocopying permitted by license only

© 2000 OPA (Overseas Publishers Association)
N.V. Published by license under the
Harwood Academic Publishers imprint,
part of the Gordon and Breach Publishing Group.
Printed in Malaysia

### T Cell Migration in Three-dimensional Extracellular Matrix: Guidance by Polarity and Sensations

PETER FRIEDL* and EVA-BETTINA BRÖCKER

*Cell Migration Laboratory, Department of Dermatology, University of Würzburg, Josef-Schneider-Str. 2. 10, 97080 Würzburg, Germany*

The locomotion of T lymphocytes within 3-D extracellular matrix (ECM) is a highly dynamic and flexible process following the principles of ameboid movement. Ameboid motility is characterized by a polarized yet simple cell shape allowing high speed, rapid directional oscillations, and low affinity interactions to the substrate that are coupled to a low degree of cytoskeletal organization lacking discrete focal contacts. At the onset of T cell migration, a default program, here described as migration-associated polarization, is initiated, resulting in the polar redistribution of cell surface receptors and cytoskeletal elements. Polarization involves protein cycling either to the leading edge (i.e. LFA-1, CD45RO, chemokine receptors, focal adhesion kinase), to a central polarizing compartment (MTOC, PKC, MARCKS), or into the uropod (CD44, CD43, ICAM- 1 and –3, β1 integrins). The function of such compartment formation may be important in chemotactic response, scanning of encountered cells, and a flexible and adaptive interaction with the ECM itself. Due to the simple shape and a diffusely organized cytoskeleton, the interactions to the surrounding extracellular matrix are rapid and reversible and appear to allow a broad spectrum of molecular migration strategies. These range from (1) adhesive and haptokinetic following i.e. chemokine-induced motility across 2-D surfaces to (2) largely integrin-independent migration predominantly guided by shape change and morphological flexibility, as seen in 3-D type I collagen matrices. Their prominent capacity to rapidly adapt to a given structural environment coupled to contact guidance mechanisms set T cell locomotion apart from slow, focal contact-dependent and more adhesive migration strategies established by fibroblast-like cells and cell clusters. It is therefore likely that, within the tissues, besides chemotactic or haptotactic gradients, the preformed matrix structure has an important impact on T cell trafficking and positioning in health and disease.

*Keywords:* T cell locomotion, three-dimensional extracellular matrix, integrins, contact guidance

*Abbreviations:* 3-D, three-dimensional, APC, antigen-presenting cell, CCR, chemokine receptor, ECM, extracellular matrix, ERM, ezrin/radixin/moesin, F-actin, filamentous actin, FAK, focal adhesion kinase, ICAM, intercellular adhesion molecule, IL, Interleukin, LFA-1, leukocyte-function antigen-1, mAb, monoclonal antibody, MARCKS, myristoylated, alanin-rich C-kinase substrate, MIP-1β, macrophage inflammatory protein-1β, MTOC, microtubule-organizing center, PKC, protein kinase C, VCAM, vascular cell adhesion molecule

* To whom correspondence should be addressed. Phone: +49–931–2012737; Fax +49–931–2012700; E-mail: Peter.fr@mail.uni-wuerzburg.de

### INTRODUCTION

The extracellular matrix provides a structural framework for leukocyte migration and localization. Following transendothelial migration, the interaction of T lymphocytes with the tissue matrix and the migration and positioning therein determine the site and the efficiency of specific immune reactions in both lymphatic as well as peripheral tissues (reviewed in: Shimizu and Shaw, 1991; Ratner, 1992; Friedl and Bröcker, 2000). In this review, the cell biology of T cell migration within the extracellular matrix is summarized with reference to T cell polarization and scanning function favoring both interaction with matrix fibers and recognition of other cells.

### SPACIAL ORGANIZATION OF THE EXTRACELLULAR MATRIX

In the body, leukocyte migration is initiated by attachment to and crawling across endothelium (Springer, 1994). It is assumed that, upon transendothelial migration, activation of cell surface receptors and engagement of the cytoskeleton are sufficient to trigger long-lasting T cell motility within the tissue (Masuyama, et al., 1992; Brezinschek et al., 1995). After successful transmigration, locomoting T cells are confronted with a network of three-dimensionally interconnected multivalent ECM ligands, predominantly consisting of a collagen fiber backbone interconnected with fibronectin, hyaluronan, and other components (Shimizu and Shaw, 1991; Ratner, 1992).

The molecular mechanisms by which cells migrate are greatly determined by the model of investigation, and it is not completely understood, which of several possible interaction strategies to extracellular matrix are indispensible to T cell migration. In the past, extensive work has addressed adhesive mechanisms involved in T cell migration across surfaces coated with isolated ligands (Hauzenberger, et al. 1994 and 1995), here referred as to "haptokinetic migration" (Maheshwari and Lauffenburger, 1998). For efficient migration across a surface, the cells must establish some kind of adhesive interaction with the underlying substrate (haptokinesis). In haptokinesis, this substrate binding is particularly provided by adhesion molecules of the integrin family or CD44, binding to their respective substrates such as fibronectin and hyaluronan (reviewed in: Hemler, 1990; Shimizu and Shaw, 1991; Friedl and Bröcker, 2000). However, it has become clear that haptokinetic migration cannot be directly transferred to cell migration in 3-D matrix environments. Three-dimensional matrices of reconstituted native collagen fibers have become a popular model to mimick interstitial tissues, either in the form of plain collagen matrices or after supplementation with other ECM components, such as fibronectin, hyaluronan, and other proteoglycans (Turley, et al. 1985, Maaser et al., 1999). Such matrices are now considered as an appropriate environment to investigate cell motility and positioning mechanisms, but also cell growth, differentiation, and stimulation by growth factors (Saltzman et al., 1992; Friedl and Bröcker, 1997 and 2000).

In resting T cells, β1 integrins expressed on the cell surface support only minimal adhesion to two-dimensional (2-D) surfaces coated with physiological interstitial matrix components such as fibronectin and collagen (Haston, et al. 1982; Sundqvist, et al. 1993). Upon contact with ECM, T cell adhesion is increased through multiple stimuli, including phorbol ester treatment, culture in IL-2, or mAb cross-linking of CD3/TCR complex, resulting in a rapid yet transient switch in integrin function from low to high avidity binding (Shimizu and Shaw, 1991; Romzek et al., 1998). In the absence of such activation signals, T cell binding to the substrate generally lacks the level of binding strength required for migratory translocation, as characterized by rapid and tortuous shape changes in the absence of significant active movement ("running on the spot"). In marked contrast, although no ligand-induced activation of β1 integrins is apparent from 2-D adhesion models, the incorporation of T cells into 3-D collagen matrices results in the spontaneous and rapid onset of long-lasting motility (Haston, et al., 1982; Schor, et al., 1983; Friedl, et al., 1994). Hence, the same biochemical substrate, collagen, provides a completely different environment for T cell-substrate-interaction and migration, depending on the spatial 2-D or 3-D properties.

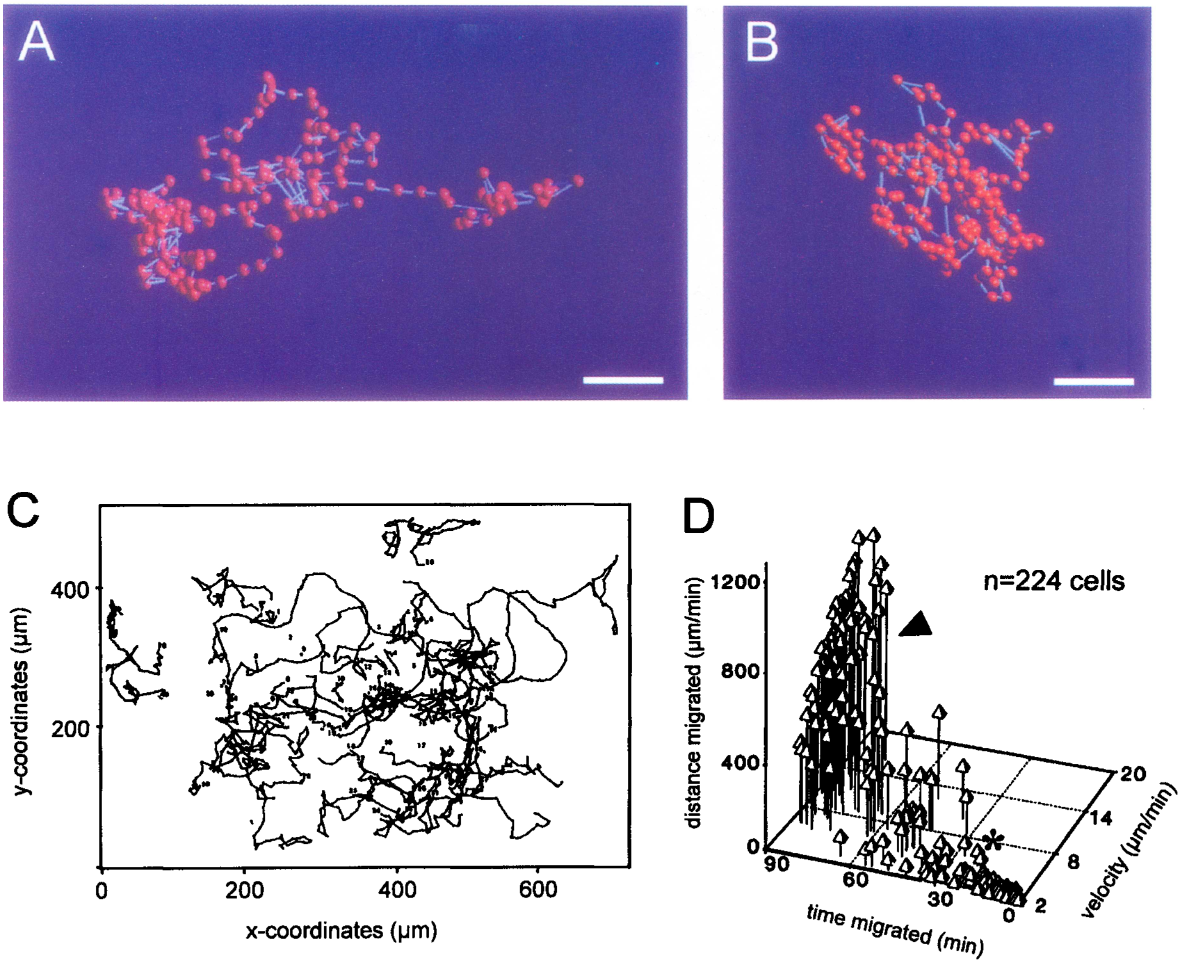

FIGURE 1 Spontaneous migration of human CD4$^+$ T lymphocytes within 3-D collagen lattices. T cells were mixed with collagen solution prior to the migration experiment. After polymerization of the matrix, migration was monitored using time-lapse- videomicroscopy and computer-assisted cell tracking, as described (Friedl et. al., 1993). (A) and (B) depict three-dimensionally reconstructed paths of 2 different cells, as obtained by automated 3-D cell tracking (Gauthier et al., 1991). The paths are composed of a rather unpredictable oscillating pattern. Linear, directionally persistent migration pattern alternates with more localized scanning behavior. (C), overview of 40 randomly selected CD4+ T cells spontaneously migrating within the field of view (63x magnification). The paths are ortotopically represented, indicating certain areas with high path density as opposed to other areas that are rarely contacted. (D), locomotor phenotypes in CD4$^+$ T cells, as represented by individual cell tracking of randomly selected cells. Data were calculated from step to step at a 1 min step interval and averaged for the entire observation period of 90 min. Maximum migration rates for individual cells range at a mean velocity of 15 µm/min in almost complete absence of stopping intervals (arrowhead). Asterisk, non-migrating population. Data represent 224 cells obtained from 4 independent experiments. Bars = 20 µm (see Color Plate XV at the back of this issue)

In 2-D models, migration requires adhesion, and perturbation of attachment, e.g. by antibodies or molecular strategies, leads to impaired migration. We will show that in a 3-D environment adhesion is not always a necessary condition for lymphocyte migration. The following considerations are important to the understanding of cell motility through 3-D extracellular matrices:

- The cells are physically surrounded by ECM ligands, hence contact to ECM is inevitable. Active adhesion mechanisms are possible, not however prerequisite for ECM contact.

- The cells are surrounded by ECM from all sides. Therefore, cell-substrate interactions and cytoskeletal organization are three-dimensionally distributed rather than unilateral to the basal side.
- Individual substrate interactions are point- or string-like, hence spatially more restricted than a ligand-coated surface, suggesting that attachment and detachment forces present at each individual interaction point in 3-D fibrous environments are lower than upon interaction with planar ligand.
- The cells must overcome biophysical resistance imposed by 3-D spatial barriers, either by proteolytic means or by morphological adaptation. This feature may not be essential for migration across 2-D substrates.
- The preformed fibrillar structure of the extracellular matrix is frequently non-random favoring contact guidance along these structures, as compared to cell crawling across a flat surface.

Taking these considerations into account, it is no surprise that the use of three-dimensional substrata reveals migration strategies in different cell types that were not predicted by 2-D haptokinetic models. As an example from a tumor cell migration model, in highly invasive melanoma cells, both α2β1 integrins and CD44 mediate migration across their respective substrates collagen and hyaluronan (Danen et al., 1993; Goebeler et al., 1996). However, if 3-D multi-component collagen substrata are used that contain hyaluronan and cross-linking chondroitin sulfate, MV3 cell migration is exclusively provided by α2β1 integrin, not, however, by CD44 function (Maaser et al., 1999). α2β1 integrin-dependent migration is neither synergized nor (after blocking of β1) replaced by CD44-hyaluronan interactions in these melanoma cells suggesting that the biophysical properties of 3-D substrata impose more restricted molecular functions of adhesion receptors, thereby limiting the predictive value of haptokinetic migration across surfaces (Maaser et al., 1999).

### THE PARADIGM OF AMEBOID MIGRATION

In general, leukocyte migration either across 2-D surfaces or within 3-D extracellular matrix shares many features of ameboid movement (Stössel, 1994). Ameboid migration ("crawling") strategies are extensively studied using the lower eukaryote *Dictyostelium disoideum* (Taylor and Condeelis, 1979), resulting in the *Dictyostelium* paradigm of ameboid cell motility. Ameboid migration can be defined as the rapid movement of simply shaped cells lacking defined cytoskeletal compartmentalization and anchoring structures towards the underlying substrate (Taylor et al., 1976; Taylor and Condeelis, 1979; Friedl and Bröcker, 2000). In *Dictyostelium*, migration is achieved by at least four sequential and interdependent processes: (1) extension of the leading margin, (2) attachment of the new extension to the underlying substratum, (3) contraction of the cell body, and (4) retraction and detachment of the trailing edge (Condeelis, 1993). On a molecular basis, ameboid movement comprises two interconnected subsystems: (1) a signal transduction system required for chemotactic responses and coupling of cell surface receptors to cytoplasmic elements and (2) the cell movement apparatus consisting of cytoskeletal proteins generating shape changes and physical forces for actual migration (Condeelis et al., 1976). The precise mechanisms orchestrating cytoskeletal dynamics into coordinated shape change and migration are current subject of investigation and summarized elsewhere (Stössel, 1994; Mitchison and Cramer, 1996; Bailly et al., 1998). As main characteristics, the migration of ameba is fast, oszillating, and highly responsive towards external stimuli, such as cAMP or chemotactic factors (Condeelis et al., 1990; Bailly et al., 1998). Cells utilizing ameboid migration strategies do not develop focal contacts or stress fibers but rather contain a diffusely organized cytoskeleton of gel-like filamentous actin (Stössel, 1994; Mitchison and Cramer, 1996). The molecular composition of adhesion sites towards the underlying substrate apparently lacks specifically enriched adhesion receptors and follows no apparent structural paradigm. In *Dictyostelium* the loss of the talin equivalent does not impair cytoskeletal dynamics and motility, although is does delay cytokinesis (Niewöhner et al., 1997). These characteristics set ameboid crawling apart from migration strategies utilized by slower cells that depend on adhesion-driven migration strategies, such as fibroblasts, tumor cells, and cell clusters (see below).

### LYMPHOCYTE MIGRATION IN 3-D EXTRACELLULAR MATRIX

Similar to ameba, leukocytes including T and B cells, neutrophils, and monocytes display a simple polarized shape, high migration velocities (in the range of several µm/min), the capacity to undergo profound shape change within seconds, and a striking lack of focal contacts and stress fibers (Stössel, 1994; Friedl et al., 1998).

Consequently, the motility of lymphocytes is extremely plastic and heterogeneous (Friedl et al., 1994 and 1995). Although virtually no adhesion and spontaneous crawling are detected *on* 2-D collagen substrate, immediate and vigorous migration is obtained *within* 3-D collagen matrices (Fig. 1). After polymerization of the collagen lattice, non-activated peripheral $CD4^+$ T cells spontaneously develop rapid and oscillating random migration, that does not display any apparent rhythmicity or persistence (Fig. 1 A, B). The tortuous paths follow no preferential directionality (Fig. 1 C) as predicted by the model of "persistent random walk" of non-directional movement. From an overview perspective, a given matrix compartment seems to be efficiently scanned (Fig. 1 C), putatively guided by ràndomly orientated collagen fibers (Friedl and Bröcker, 2000a). Only 20 to 50% of the population develops spontaneous migration (Fig. 1D, arrowhead), whereas the remainder subset shows only temporary activity or a sphercical non-migratory state (Fig. 1 D, asterisk). An average step length of 7 µm/min and peak velocities reaching 20 µm/min (Fig. 1D, arrowhead) implicate efficient and sophisticated matrix-binding and -detachment strategies within the collagen network.

### INDUCTION OF MIGRATION

Upon transition from sessile and round morphology to a migratory state, cells acquire a polarized morphology. In ameba, pseudopod extension at the leading edge is a hallmark of cell movement, determining cell polarity and direction of locomotion during chemotaxis (Condeelis et al., 1976). In T cells, polarization is a uniform reaction to multiple different stimuli, such as contact with native or denatured extracellular matrix (Friedl et al., 1994), activating cytokines and chemokines (Friedl et al., 1995; Wilkinson and Newman, 1992), infection with intracellular bacteria (Arencibia et al., 1997), as well as contact with other cells, such as endothelium (Springer, 1994). In addition, T cell migration in collagen lattices is a consequence of activation, either by cross-linking mAbs against CD3 and CD2 (Nikolai et al., 1998), mitogenic stimuli (Wilkinson, 1986), or the presence of dendritic cells (Gunzer et al., 2000).

On a molecular basis, several signal transduction pathways appear to converge towards the induction of T cell polarization and migration. These comprise cross-linking of α4 integrins (Friedl et al., 1995; Hauzenberger et al., 1997), LFA-1, ICAM-3, or CD43 (Nieto et al., 1997), activation of protein kinase C by phorbol esters (Islam and Wilkinson, 1992; Entschladen et al., 1997), tyrosine phosphorylation of focal adhesion kinase and other proteins (Shaw et al., 1995; Entschladen et al., 1997), or expression of a constitutively activate mutant of Rac (D'Souza-Schorey et al., 1998). Integrin-triggered migration involves tyrosine phosphorylation of the focal adhesion kinase (Shaw et al., 1995; Hauzenberger et al., 1997; Entschladen et al., 1997), whereas chemokine receptor pathways are coupled to pertussis toxin-sensitive G proteins and protein kinase C (Nieto et al., 1996; Vicente-Manzanares et al., 1998). Similarly, inhibition of Rho activity by *Clostridium botulinum* exoenzyme C3 results in polarization of previously spherical Jurkat T cells (Woodside et al., 1998), implicating Rho in the maintenance of a sessile state counter-balancing migration. Several other signaling cascades are interconnected with integrin function in T cells, putatively counteracting migration. During the process of antigen presentation, signals induced by accessory molecules, such as CD2 and CD28, can induce activation of PI-3-kinase and tyrosine phosphorylation of Cbl, ultimately resulting in engagement of β1 and β2 integrins and firm adhesion to ECM and cellular ligands (Shimizu and Shaw, 1991; Dustin et al., 1997; Zell et al., 1998; Kivens et al., 1998). These and similar events are thought not only to favor TCR triggering and T cell activation, but also to achieve a sessile, adhesive state for stable cell-cell interaction (Shimizu and Shaw, 1991; Dustin, 1997).

### TOPOLOGY OF MIGRATION-ASSOCIATED POLARIZATION

In 3-D collagen matrices, migrating T cells develop a highly polarized, "hand-mirror shaped" morphology (Fig. 2): a leading edge, the main cell body, and the trailing edge, consisting of a small cytoplasmic backword projection (Haston et al., 1982; Wilkinson, 1986; Sanchez-Madrid and del Pozo, 1999).

### THE LEADING EDGE

The leading edge is usually broad and highly dynamic consisting of several small ruffling filopodia and one or two protruding lamellipodia (Fig. 2). This is the most dynamic zone of the migrating T cell containing focal adhesion kinase, an enzyme required for cytoskeletal dynamics at substrate interactions and the turn over of focal contacts (Ilic et al., 1995). Cell adhesion receptors involved in contact with other cells, such as LFA-1 (Friedl et al., 1998) and CD45RO (Fig. 3A) frequently accumulate at the leading edge. Additionally, in activated but not in resting cells, chemokine receptors, such as CCR2, a receptor for MCP-1, –3, and –4, and CCR5 binding RANTES and MIP-1β were detected at the leading edge of crawling T cells (Nieto et al., 1997).

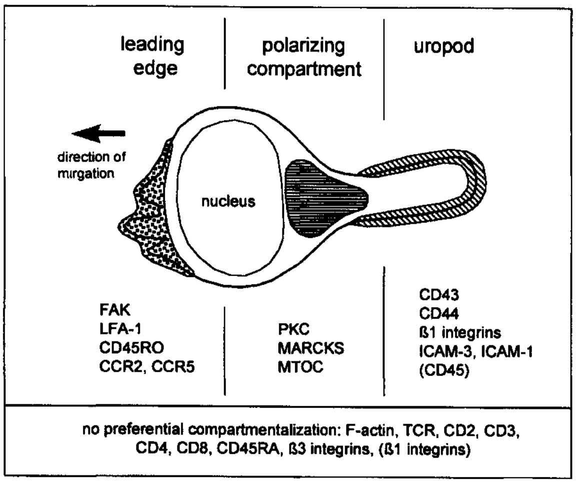

FIGURE 2 Topographic representation of migration-associated T cell polarization. Data are summarized for 2-D and 3-D migration as well as transendothelial migration models

### THE "POLARIZING COMPARTMENT"

The main body is round-shaped and contains the nucleus followed by a narrowing transition zone, that might function as a "polarizing compartment" (Fig. 2) generating the transition from the cell body towards the uropod. Here, the microtubule-organizing center (MTOC) is localized (Ratner et al., 1997) as well as protein kinase C (PKC) (Entschladen et al., 1997) in colocalization with the PKC-substrate MARCKS (F. Entschladen and P. Friedl, unpublished observation). Although the precise function of this compartment remains to be established, MARKS represents a putative link from PKC-dependent activation pathways (such as chemokine-induced T cell migration) and the actin cytoskeleton (Myat et al., 1997).

### THE UROPOD

The trailing edge is formed by a specialized cytoplasmic projection of approx. 3 to 5 μm in length and 1 to 3 μm in diameter, termed the uropod (Haston et al., 1982; del Pozo et al., 1998). The cytoplasmic portion of the uropod contains multiple cytoskeletal elements such as F-actin, radixin, moesin, and tubulin (Ratner et al., 1997; del Pozo et al., 1997). Strikingly, the plasma membrane of the uropod is rich in adhesion receptors. These include CD44, the principal receptor for hyaluronan and CD43 (leukosialin), a putative anti-adhesive molecule involved in cell detachment (Sanchez-Mateos et al., 1995), as well as β1 integrins, ICAM-3, and ICAM-1 (Friedl et al., 1998; Serrador et al., 1997; del Pozo et al., 1998). In T cells migrating in 3-D collagen lattices, other receptors are occasionally seen in the uropod such as LFA-1 and CD45 (P. Friedl, unpublished). It is conceivable that adhesion molecules are directed by cytoskeletal linkages. In polarized T cells, moesin is co-immunoprecipitated with ICAM-3, CD44, and CD43 suggesting that the redistribution of these components is dependent on the actin cytoskeleton (Serrador et al., 1997). Moesin is a member of the ezrin-radixin-moesin family of closely related adapter proteins that physically interact with the actin cytoskeleton and also mediate signal transduction (Tsukita and Yonemura, 1997). The uropod was recently described as an important and highly specialized cell compartment of putative adhesive function and interaction of cell surface receptors with the cytoskeleton (del Pozo, 1998; Sanchez-Madrid and del Pozo, 1999). In 3-D collagen, the uropod has an important stabilizing and anchoring function in migrating T cells (see below). Furthermore, in T cells adhering to endothelium or other 2-D substrata, the uropod may capture other leukocytes and mediate homotypic aggregation via adhesive mechanisms. Therefore, it was proposed that the uropod acts as a cell recruitment instrument and amplificator of transendothelial migration (del Pozo et al., 1996 and 1997).

No preferential accumulation to either compartment is seen for TCR and associated molecules such as CD3, CD4, CD2, and CD45RA (Fig. 3B), as well as for β3 integrins, receptors for IL-2, TGF-β, and TNF-α, and, in some cells, β1 integrins (Nikolai et al., 1998; Friedl et al., 1998), suggesting the absence of constitutive migration-associated cytoskeletal linkages in these receptors. F-actin forms a diffuse linear subcortical layer without apparent stress fibers or association with focal contacts (Friedl et al., 1998), as previously described for other ameboid cells (Stössel, 1994).

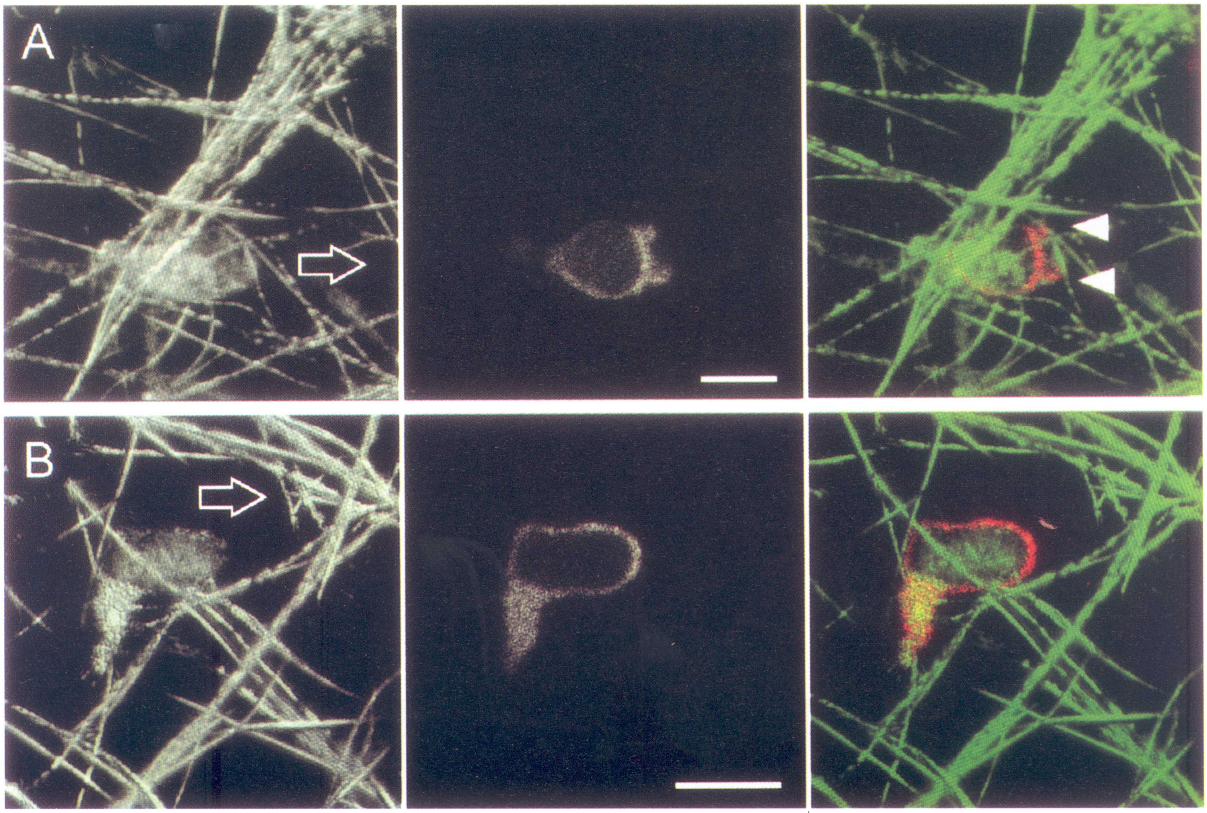

FIGURE 3 Differential redistribution of CD45RO (A) and CD45RA (B) on spontaneously migrating CD4+ T cells within a 3-D collagen matrix. Confocal reflection of the matrix structure (left) was reconstructed for 3 consecutive scans of ± 1 μm from the center of the cell. Arrows indicate the direction of migration. Confocal immunofluorescence was obtained after fixation and staining with anti-CD45 antibodies and secondary Fab-fragments (center). Superimposition of matrix structure and immunofluorescence (right) shows preferential accumulation of CD45RO at the leading lamella at newly forming interaction sites to collagen fibers. In contrast, CD45RA is randomly distributed over the entire cell body. The direction of migration is indicated by arrows. Bars = 5 μm (see Color Plate XVI at the back of this issue)

The differential compartment formation in polarized and/or migrating T cells, here referred to as "migration-associated polarization", appears to be an inherent default program seen under many different conditions, that are associated with the onset of migratory induction. As outlined above, these include binding to and crawling through endothelium, migration within ECM, response to chemotactic factors and, to some extent, the binding to resident cells (del Pozo et al., 1997 and 1998; Entschladen et al., 1997). Migration-associated polarization, however, is distinct from more static, cell contact-dependent polarization upon specific binding to antigen-presenting cells. These interactions lead to clustering and capping of T cell receptor, CD4 and LFA-1 together with cytoskeletal and signaling proteins towards the cell-contact plane to antigen-presenting cells (Monks et al. 1998; Shaw and Dustin, 1997; Dustin et al., 1998).

### FUNCTIONS OF MIGRATION-ASSOCIATED POLARIZATION

Although the precise function of spatial asymmetry of cell surface receptors and cytoskeletal elements requires further investigation, several important cell function have emerged recently as to be dependent on this polarization program. These include the chemotactic response (Nieto et al., 1997), cell-cell (del Pozo et al. 1996), and cell-matrix interactions (Friedl et al., 1998).

First, cell migration is induced and directed by chemical attractant gradients and requires sensory asymmetry of the cell. Polar redistribution of chemokine receptors together with adhesion molecules (e.g. LFA-1) towards the leading edge is one likely mechanism by which a chemotactic signal might be detected and integrated into a cytoskeletal response (del Pozo, 1998). Soluble or immobilized gradients of promigratory factors could dominate signal transduction preferentially at the leading edge, favoring polarized cytoskeletal activity towards the source of these chemotactic factors (Gilat et al., 1994; Bolognini et al., 1986; del Pozo, 1998).

Second, polarization is prerequisite for efficient sensing, scanning, and grasping of target cells. Migrating T cells are polarized antigen sensors for efficient interaction with antigen-presenting cells (Negulescu et al., 1996). Upon contact with antigen-presenting B cells, the leading edge of crawling T cells is approximately four-fold more sensitive to T cell receptor-mediated triggering of $Ca^{++}$ influx than the uropod (Negulescu et al., 1996). LFA-1 at the leading edge is a candidate receptor for initial scanning activity upon cell encounters. After TCR triggering, high avidity binding of LFA-1 to ICAM-1 is induced, mediating firm attachment to the APC and immobilization of the T cell (Dustin et al., 1997).

Third, migration-associated polarization is required for graded interaction with the extracellular matrix itself, allowing sensing of collagen fibers, transient anchoring and efficient translocation (Friedl and Bröcker, 2000).

### ADHESION RECEPTORS IN T CELL MIGRATION WITHIN EXTRACELLULAR MATRIX

### Haptokinetic T cell migration

β1 integrins (Hemler, 1990) are essential for migration of T cells across ligand-coated surfaces. Integrins α1β1, α2β1, and α3β1 predominantly bind to collagen, α4β1 and α5β1 bind to fibronectin, and α6β1 is the principal receptor for laminin (Hemler, 1990). Additionally, αvβ3 was shown to interact with multiple ECM ligands, among them vitronectin and denatured collagen. T cell migration across or towards fibronectin is reduced by antibodies directed against integrins α4 and α5 chains, or the common β1 chain (Hemler, 1990; Hauzenberger et al., 1994 and 1995; Gilat et al., 1994; Arencibia et al., 1997), while migration across laminin is impaired by anti-α6β1 integrin mAb or deletion of the α6 cytoplasmic domain (Gimond et al., 1998). Likewise, migration of activated T cells across type I collagen appears to partial depend on β1 integrins (Sundqvist et al., 1993 and

1993a), although further identification of α chains is lacking.

Other non-ECM adhesion pathways such as LFA-1/ICAM-1 (Dustin et al. 1992), the CD2/CD58 interactions (Dustin 1997a), or α4β1/VCAM-1 (Springer, 1994) were shown to mediate haptokinetic migration, if the respective counterpart ligand is present.

Adhesion receptor function in haptokinetic T cell migration may correspond to migration biophysics established for fibroblasts. In haptokinetic migration of fibroblasts, the interdependence of adhesion strength and motility rate are a function of both receptor density and activation state as well as ligand density (Palecek et al., 1997). Optimal migration rates are obtained at intermediate adhesion strength allowing both, attachment (at the front) and detachment (of the trailing edge) as a cycling process (Lauffenburger and Horwitz, 1996). If the cells develop high binding strength, however, the reduction of attachment by e.g. mAb will shift the cells towards intermediate adhesion strength, yielding increased migration, as sometimes seen in fibroblasts or neurons (Lauffenburger and Horwitz, 1996; Palecek et al., 1997).

In resting and a major subset of activated T cells binding sites of the β1 family remain locked in the default low affinity state (Faull et al., 1994) and adhesion strengh to most substrata is low (Hemler, 1990). Triggering T cells by phorbol esters results in integrin redistribution towards attachment sites in the absence of affinity changes (Faull et al., 1994). As a consequence, at low to intermediate binding strength, impairing T cell adhesion to substrate means reducing attachment and, hence, motility rates. In contrast to fibroblasts, in T cells the receptor of investigation could either be directly involved in the migratory action or, alternatively, act as an unrelated "sticky" bystander molecule, favoring ameboid movement by preventing cell drifting by media convection (the phenomenon of drifting which is regularly seen in 2-D liquid cultures of T cells). In the second case, the contribution of the receptor to cell migration would be indirect.

### T cell migration in 3-D matrices

In contrast to haptokinetic migration across surfaces, migration of T cells in solid tissues is a complex process with several possible cell-matrix and cell-cell interactions. Due to its complexity, progress in identifying adhesion receptors involved in 3-D migration models has been slow. Initial observations suggested the involvement of an unidentified collagen binding receptor in T cell motility (Arencibia and Sundqvist, 1989) and β1 integrin function in T cell penetration of 3-D collagen lattices (Sundqvist et al., 1993). However, using more sensitive continuous-time cell-tracking approaches, the migration of resting T cells as well as of concanavalin A-activated T blasts was not affected by antibodies blocking different adhesion-related epitopes on β1 integrins (Friedl et al., 1998). In 3-D collagen, collaboration of other integrins appears unlikely, as shown by simultaneously blocking β1, β2, β3 and αv chains. Neither the number of migrating cells, their individual velocities nor the path structures were affected by these antibodies (Friedl et al., 1998). Given the requirement of integrin functions in 2-D migration models, the lack of detectable integrin function in spontaneous T cell motility through collagen matrices is unexpected, raising the possibility that a certain degree of "non-specific" biophysical interaction is present in ameboid motility through 3-D tissue.

We have recently described, that the migration of different cells is not a uniform process but rather includes different cellular and molecular mechanisms, depending on functional states of the cell and the environment encountered. (Friedl et al., 1998a and 1998b). Based on this model, T cells might be able to develop a range of several different strategies for translocation (Fig. 4): depending on the size and rigidity of the cell body, expression level and functional state of expressed integrins, and the biophysical properties of the extracellular matrix encountered, T cells could either use integrin-independent or integrin-dependent migration strategies.

If a 3-D substrate provides little "specific" stickiness lacking ligand-induced adhesion receptor engagement and clustering via signaling and

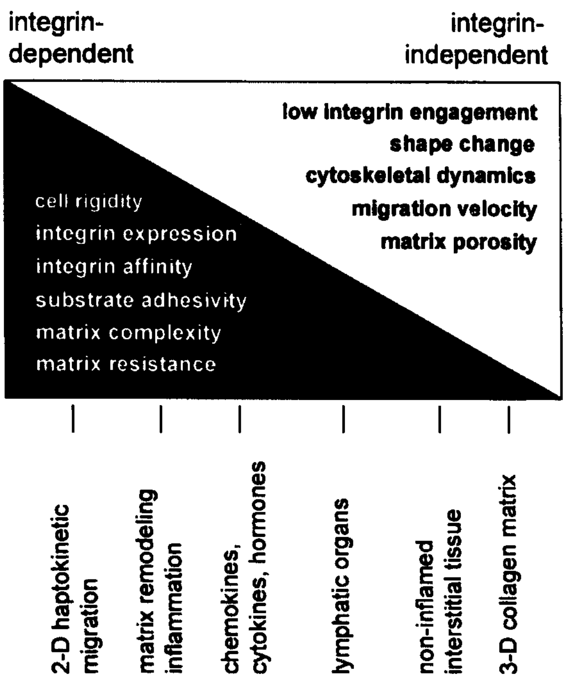

FIGURE 4 Versatility in T cell migration – a model cytoskeletal linkages (Miyamoto et al., 1999), low affinity interactions in conjunction with shape change might be sufficient as to mediate a certain degree of migration. Such binding could be provided by little defined charged residues such as carbohydrates or by hydrophobic bonds, supporting a sufficient degree of contact rigidity to ECM components. The notion of "biophysical" migration strategies in 3-D collagen substrata was initially proposed by Haston et al. (1982), based on electron microscopic images of T cells simply "wrapping" filopodia and membane protrusions around collagen fibers (Haston et al., 1982). Similarly, the migration of neutrophils within the loose collagen network of amniotic membrane appear to be guided by physical events, such as lateral protrusions ("footholds") and constriction rings caused by narrow matrix pores (Mandeville, et al. 1997). Theoretically, propulsion of the cell body could occur just by shape change independent on specific adhesion (Fig. 4). Such "biophysical" migration strategies could dominate non-activated T cells of low integrin-expression in tissues of large enough porosity and low adhesivity, e.g. in 3-D lattices of native collagen fibers devoid of additional ECM components, non-inflamed reticular tissues, or within the loose fibrous network of lymphatic organs.

On the other hand, larger cell size and higher integrin expression in long-term activated T-blasts (i.e. many cell lines used for motility studies) could favor the utilization of receptor-driven mechanisms. Receptor-dependent migration is likely to occur in tissues providing sufficient adhesion sites, e.g. upon chronic inflammation, in the presence of activating cytokines, chemokines, and hormones (Taub et al., 1994), or on 2-D substrates.

Chemokines such as MIP-1β and RANTES, either as soluble factors or bound to ECM, induce β1 integrin-dependent T cell adhesion to a variety of substrates, including collagen, fibronectin, laminin, or basement membrane equivalent, mediated by pertussis toxin-sensitive mechanisms (Gilat, et al., 1994; Lloyd et al., 1996). It is likely, that chemokines initiate migration via two interdependent mechanisms, (a) by inducing a signaling cascade activating the cytoskeleton and (b) by intensifying the contact to ECM via adhesive mechanisms. Blocking of either pathway inhibits migration. In T cells stimulated by cytokines (e.g. IL-2) or chemokines (e.g. IL-8 or RANTES), the migratory induction is reduced by antibodies blocking α2, α4, or α6 integrins, whereas the baseline migration rate remains unaffected by these antibodies (Friedl et al., 1995; Franitzka, 1999). These findings underline the versatility of T cell migration mechanisms in different activation states and migration models.

It is known that leukocyte migration within tissues is an extremely robust process. In neutrophils migrating through the stroma of the rat mesentery in vivo, the initial migration velocity of 12 μm/min is reduced to a mean of 5 μm/min in the presence of blocking β1 integrin antibodies, while no additional effect is seen by blocking β2 integrins (Werr et al., 1998). Residual leukocyte migration comprising individual cell velocities ranging from 2 to more than 10 μm/min is present in virtually every 3-D migration model after receptor blocking (Werr et al., 1998; Friedl et al.,

1995; Franitzka et al., 1999). Conceptually, residual migration could either represent the action of alternative adhesion receptor systems, however, could also support the concept of biophysical migration strategies that are insensitive to antagonizing specific adhesion sites (Friedl and Bröcker, 2000).

Beyond the scope of this model, bystander or target cells present in the tissue could modulate molecular migration requirements in T cells. Although integrins are the best-studied family of adhesion receptors, immunoglobulin- and cadherin family members also mediate cell migration. T cell migration into solid spheroids composed of glioma cells and a sparse fibronectin-containing extracellular matrix, mimicking the tumor intercapillary microenvironment of glioma tumors, was most significantly inhibited by perturbation of the LFA-1/ICAM-1 adhesion pathway, but not by broadly blocking anti-β1 integrin mAb, suggesting that LFA-1/ICAM-1-mediated crawling contacts to glioma cells dominated over fibronectin-dependent cell-matrix interactions in these spheroids (Jääskeläinen et al., 1992).

### MIGRATION STRATEGIES IN OTHER CELLS

The possibility to develop such a diverse range of dynamic and volatile interactions in different environments sets migrating leukocytes apart from other cells of high default integrin expression and strong integrin-driven substrate adhesion, such as fibroblasts, keratinocytes, and many tumor cells (Friedl et al., 1998b). In those cells, cell migration is dependent on defined force-generating extracellular matrix-integrin complexes, termed focal contacts (reviewed in: Sheetz et al., 1998; Lauffenburger and Horwitz, 1996; Burridge and Chrzanowska-Wodicka, 1996). The migration of these cells is slow (0.1 to 2 μm/min), dependent on integrin clustering at interactions to collagen fibers, and integrin-mediated adhesion (Maaser et al., 1999). In these cells, migration-associated traction and reorganization of the collagen fibers are coupled to the release of cell surface receptors from the trailing edge and deposition into the matrix (Friedl et al., 1997). All of these functions can be blocked by antagonizing integrin-mediated adhesion, indicating that integrin-mediated attachment is indispensible for this migratory prototype (Maaser et al., 1999). Differences in migration strategies detected for the same 3-D collagen matrix migration model implicate a variety of biochemical and biophysical properties in cells of different origin (reviewed in Friedl et al., 1998a and 1998b) but also, as proposed here for T cells, for the same cell type, depending on its integrin expression and activation state, and the tissue context of migration (Fig. 4).

### SENSORY FUNCTIONS AND CONTACT GUIDANCE

As monitored by continuous-time confocal reflection microscopy (Friedl and Bröcker, 2000a), the biophysical events in T cell migration can be observed at high detail resolution, here represented as the 3-D reconstruction of a spontaneously migrating $CD4^+$ T cell (Fig. 5). In the process of migration, the main body is usually positioned parallel to one or a few directionally "dominant" fiber strands (Fig. 5 A, black arrowhead). The ruffling leading edge constantly develops new interactions, resulting in forward movement of the leading edge along one or a few fiber strands (Fig. 5 B, arrowhead), frequently in parallel orientation. Upon binding, minor fiber bending and traction are sometimes observed. Simultaneously, the uropod continuously interacts with one or several fibers transiently anchoring the cell in its 3-D position (Fig. 5 A, white arrowhead). Detachment of the uropod appears to occur as a passive process, putatively resulting from a gradient of low forces from the leading to the trailing edge, as represented by slight fiber bending towards the detaching cell body (Fig. 5 A, arrowhead). Upon cell translocation, uropod-bound fibers detach and bounce back into their previous position, as detected by confocal reflection time series (Friedl et al., 1998; P. Friedl, unpublished).

T cell positioning along preformed matrix structures was previously described by transmission light

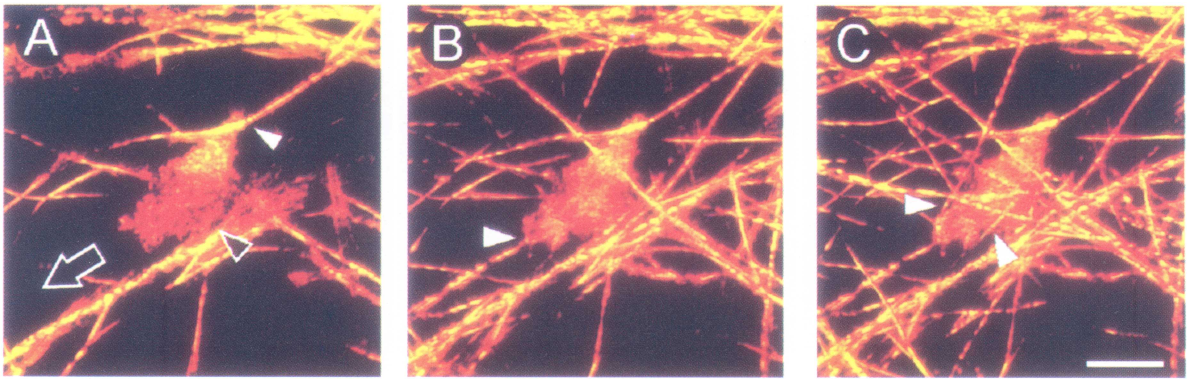

FIGURE 5 Biophysics of T lymphocyte migration in a collagen lattice: three-dimensional reconstruction by confocal reflection microscopy. (A), single central scan showing the direction of migration (arrow), alignment of the cell body parallel to a fiber strand (black arrowhead), and the transient anchoring of the uropod to one fiber that is pulled towards the cell body upon forward movement of the cell (white arrowhead). The dynamics described herein were confirmed by confocal time-series scanning on non-fixed cells in the process of migration (Friedl et al., 1998). (B), reconstruction of the lower portions of cell and matrix showing the leading edge in contact with an underlying guiding fiber (white arrowhead). (C), additional reconstruction of the top sections depicting a narrow pore at the leading edge through which the cell body begins to flow (white arrowheads). Bar = 5 µm (see Color Plate XVII at the back of this issue)

microscopy for ex vivo lymphocyte movement in the connective tissue of rat mesentery, showing guided movement along preexisting structures such as collagen bundles (Haemmerli et al., 1983). Non-reorganizing attachment and detachment strategies are also present in neutrophils (Mandeville et al., 1997) as well as dendritic cells ( et al., 1997), and appear to correspond to contact guidance as an important motility mechanism along preformed physical structures, here collagen fibers (Wilkinson et al., 1983; Shields et al., 1984; Guido et al., 1993).

The forces generated by crawling leukocytes are relatively week, compared to e.g. fibroblasts. In neutrophils, the extension and force production of the leading lamella is a periodic process at 0.01 to 0.03 Hz, generating forces in the range of tenths of µdyn, as measured from the retention force of membrane-bound magnetic beads using a magnetic force transducer (Guilford et al., 1995). In comparison, forces generated by fibroblast ruffles are at least ten-times higher (15 to 30 µdynes) (Felder and Elson, 1990; Elson et al., 1999), correlating well with their powerful collagen fiber bundling and reorganizing capacity during migration (Friedl et al. 1998b).

It can be speculated that, because of their low forces, migrating T cells must adapt their morphology to the preformed environment, squeezing the cell body through preexisting matrix gaps (Fig. 4C, arrowheads, as detected by sequential 3-D reconstruction) rather than structurally changing the matrix architecture. Increasing the biophysical resistance, i. e. using higher collagen concentration (2,5 mg/ml) and concomitantly decreasing pore size (Reid and Newman, 1991; Friedl et al., 1997) strongly reduces motility. The concept of morphological flexibility and shape change as important mechanism to overcome biophysical matrix resistance is consistent with the finding that microtubule inhibitors induce T cell migration in collagen (Wilkinson, 1986; Ratner et al., 1997; Nikolai et al., 1999), presumably by increasing deformability of the cell body facilitating migration through constricted spaces (Ratner et al., 1997). Conversely, the stabilization of microtubule polymers appears to counteract lymphocyte migration supposedly by reducing morphological flexibility (Wilkinson, 1986). Further support for the morphological adaptation theory comes from the observation, that T cell motility in 3-D collagen lattices does not generate collagen digestion fragments (Schor et al., 1983) nor lysis or contraction of the preformed matrix structure (P. Friedl, personal observation). In this context, it will be important to determine under which circumstances proteolytic remodeling of the interstitial matrix, provided by matrix metalloproteinases, is involved in T cell migration (Leppert et al., 1995).

In summary, similar to ameba, T cells utilize rapid and highly dynamic low-adhesive migration strategies, that are dominated by shape change and morphological adaptation rather than structural reorganization of the matrix. These features open up the possibility, that, besides chemotactic gradients, matrix paths present in the tissues can guide and direct T cell trafficking once the cells have penetrated the basement membrane within a given compartment.

### LEUKOCYTE MIGRATION WITHIN IN VIVO TISSUES

In lymphoid and non-lymphoid tissues, the structure of the extracellular matrix is non-random resulting in fine compartmentalization of the ECM ultra structure. In lymphatic organs, T cell zones and lymphocyte traffic areas contain an extensive number of extracellular fibers, referred to as "reticular fibers" because of their reticular pattern. The structure of the reticular fiber network in T cell zones in mouse lymph nodes, spleen, and Peyer's patches is a highly organized "open" network, mainly composed of collagen types I and III as well as fibronectin, collagen type IV, and laminin (Gretz et al., 1996; Ohtsuka et al., 1992). The texture of this compartment is a loose and spacy strand-like fibrous network that is conserved in orientation at pore sizes ranging from 3 to 15 μm, sometimes resembling to channel-like structures. In the lymph node, fibers strands forming channels ("conduits") are oriented towards the follicular B cell zones (Gretz et al., 1997). In Peyer's patches, fiber strands converge towards the follicle domes, the area of antigen presentation (Ohtani et al., 1991; Ohtsuka et al., 1992). Hence, it was speculated that T cell trafficking pathways from the periphery to the locations of antigen-presentation is guided by the preformed reticular fiber network (Ohtsuka et al., 1992). A similar situation is found in the skin. In the dermis, solid non-random collagen fiber strands alternate with path-like structures containing glycosaminoglycans of little solid texture. After fixation and sample preparation, these zones impose as gap-like shrinking artifacts of little matrix texture, particularly if additional edema is present (Fig. 6, arrowheads). In atopic dermatitis, large amounts of T cells triggered by unknown mechanisms transiently infiltrate the upper dermis via non-destructive mechanisms (Fig. 6 A, "T"). At high magnification, the inflammatory infiltrate frequently shows polarized leukocytes in stand-like order aligned in between solid fiber strands within matrix gaps (Fig. 6 B-D, arrowheads), suggesting that migration occurred along these matrix structures. In the dermis loose reticular networks are present in proximity to the epidermal basement membrane (Fig. 6 B), the papillae (Fig. 6 C), and along blood vessels (Fig. 6 D). These preformed gaps, cords, or channels appear as ideal substrate for ameboid migration and contact guidance preventing random walk (Wilkinson et al., 1983). Contact-mediated migratory persistence may occur in the presence or even absence of stable chemotactic gradients and the spaciousness of such pathways might favor high migration velocities independent on the proteolytic capacity of the cells.

### PERSPECTIVES

Understanding the interplay of physical and biochemical migration strategies on the one hand, and the impact of the tissue structure on the other hand will be crucial to better dissect migration and positioning mechanisms in host defense and autoimmune disease. It is conceivable that pathologic deviation from a highly organized, "default" matrix structure caused by proteolytic matrix remodeling may interfere with the molecular events and efficiency of leukocyte migration. For T cell tafficking, both entrance as well as exit of a given tissue compartment might be regulated by matrix structures, resulting in either T cell depletion or accumulation in the tissue. In the case of

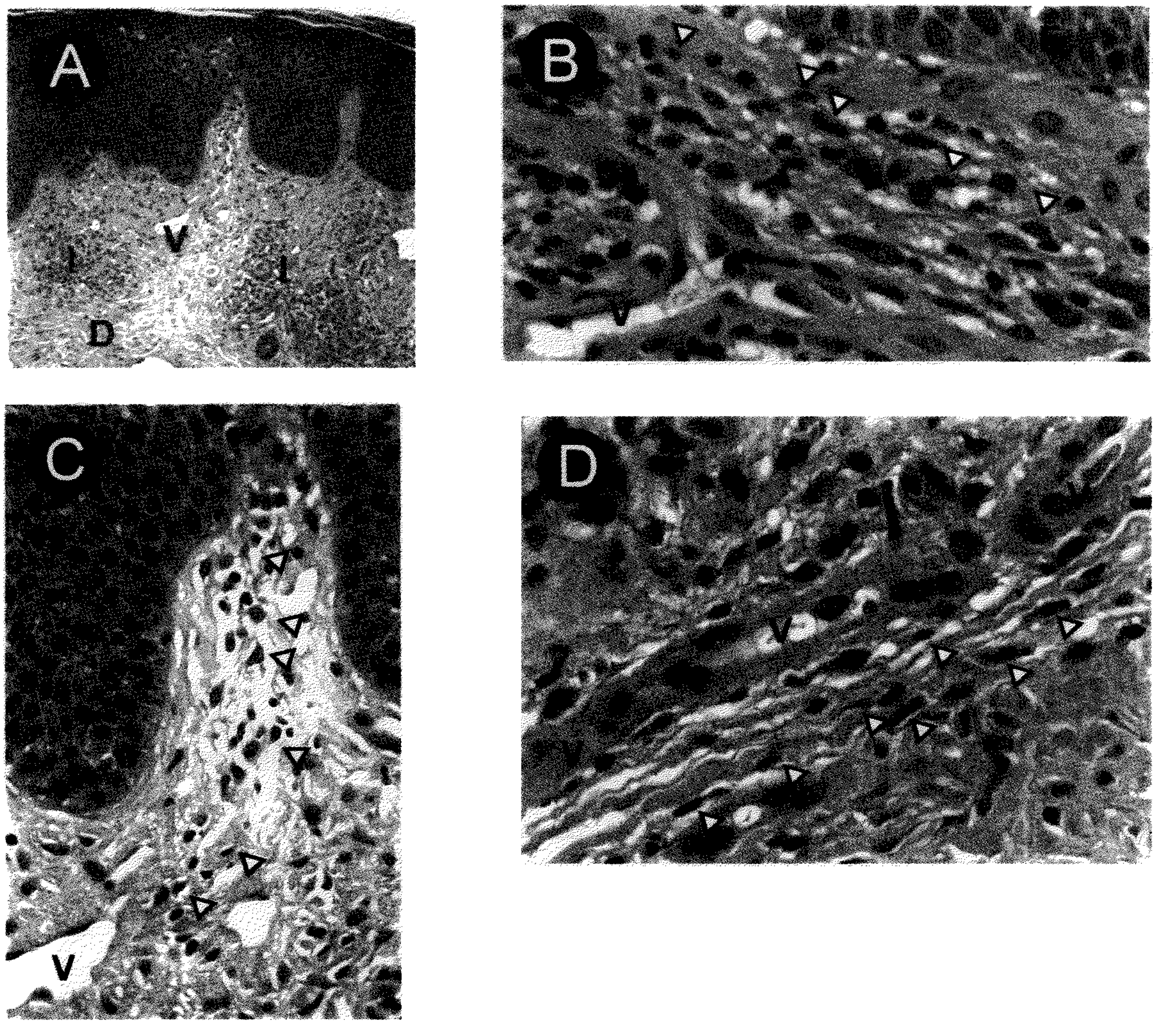

FIGURE 6 Leukocyte trafficking pathways in a non-destructive inflammatory reaction (atopic dermatitis). (A), overview of epidermis (E) and dermis (D) containing vessels (V) and a mixed leukocytic infiltrate (I) in the upper dermis, predominantly constisting of lymphocytes. (B), reticular network of parallel fibers in close proximity to a blood vessel containing passenger leukocytes in cord-like order (white arrowheads). (C) Leukocyte trafficking pathway (arrowheads) from a vessel in the upper dermis towards the tip of a dermal papilla. (D) Loose reticular fibrous network in parallel orientation along a blood vessel infiltrated by multiple polarized leukocytes (arrowheads) along these fiber strands (as indicated by elongated shape of the nucleus). H & E stain of tissue sections obtained from a patient with chronic atopic dermatitis, a non-destructive inflammatory dermo-epidermal inflammatory disorder involving lymphocytic infiltration of the upper dermis and epidermis wound healing, a loose fibrin network is temporarily produced favoring cell motility. On the other hand, in tumor-induced matrix remodeling, removal of preexisting matrix gaps might impede T cell migration (Applegate et al., 1990) and impose a bias for previously activated T cells acquiring proteolytic means for tissue penetration (Leppert et al., 1995). In further studies, 2-D and 3-D ECM in vitro models in conjunction with in vivo trafficking studies will help to establish the basis of different cell migration strategies in health and disease as a prerequisite for differential adhesion receptor targeting.

### Acknowledgements

This project was supported by a grant from the Deutsche Forschungsgemeinschaft (Fr 1155–2/1). We gratefully acknowledge Dr. C. Rose for expert help in histology.

### References

Applegate, K. G., C. M. Balch, and N. R. Pellis, N. R. (1990) In vitro migration of lymphocytes through collagen matrix: arrested locomotion in tumor-infiltrating lymphocytes. Cancer Res. 50:7153–7158.

Arencibia, I. and K.G. Sundqvist. (1989) Collagen receptor on T lymphocytes and the control of lymphocyte motility. Eur. J. Immunol. 19:929–934.

Arencibia, I., N.C. Suarez, H. Wolf-Watz, and K.G. Sundqvist. (1997) Yersinia invasin, a bacterial beta1-integrin ligand, is a potent inducer of lymphocyte motility and migration to collagen type IV and fibronectin. J. Immunol. 159:1853–1859.

Bailly, M., J.S. Condeelis, and J.E. Segall. (1998) Chemoattractant-induced lamellipod extension. Microsc. Res. Tech. 43:433–443.

Bailly, M., L. Yan, G.M. Whitesides, J.S. Condeelis, and J.E. Segall. (1998a) Regulation of protrusion shape and adhesion to the substratum during chemotactic responses of mammalian carcinoma cells. Exp. Cell Res. 241:285–299.

Brezinschek, R.I., P.E. Lipsky, P. Galea, R. Vita, and N. Oppenheimer-Marks. (1995) Phenotypic characterization of CD4+ T cells that exhibit a transendothelial migratory capacity. J. Immunol. 154:3062–3077.

Burridge, K. and Chrzanowska-Wodicka, M. (1996) Focal adhesions, contractility, and signaling. Annu. Rev. Cell Dev. Biol. 12:463–519.

Condeelis, J.S., D.L. Taylor, P.L. Moore, and R.D. Allen. (1976) The mechanochemical basis of amoeboid movement. II. Cytoplasmic filament stability at low divalent cation concentrations. Exp. Cell Res. 101:134–142.

Condeelis, J., A. Bresnick, M. Demma, S. Dharmawardhane, R. Eddy, A.L. Hall, R. Sauterer, and V. Warren. (1990) Mechanisms of amoeboid chemotaxis: an evaluation of the cortical expansion model. Dev. Genet. 11:333–340.

Condeelis, J. (1993) Life at the leading edge: the formation of cell protrusions. Annu. Rev. Cell Biol. 9:411–44:411–444.

D'Souza-Schorey, C., B. Boettner, and L. Van Aelst. (1998) Rac regulates integrin-mediated spreading and increased adhesion of T lymphocytes. Mol. Cell Biol. 18:3936–3946.

Danen, E. H. J., van Muijen, G. N. P., van de Wiel-van Kemenade, E., Jansen, K. J. F., Ruiter, D. J., and Figdor, C. G. (1993) Regulation of integrin-mediated adhesion to laminin and collagen in human melanocytes and in non-metastatic and highly metastatic human melanoma cells. Int. J. Cancer 54:315–321.

del Pozo, M.A., P. Sanchez-Mateos, and F. Sanchez Madrid. (1996) Cellular polarization induced by chemokines: a mechanism for leukocyte recruitment? Immunol. Today 17:127–131.

del Pozo, M.A., C. Cabanas, M.C. Montoya, A. Ager, P. Sanchez-Mateos, and F. Sanchez-Madrid. (1997) ICAMs redistributed by chemokines to cellular uropods as a mechanism for recruitment of T lymphocytes. J. Cell Biol. 137:493–508.

del Pozo, M.A., M. Nieto, J.M. Serrador, D. Sancho, M. Vicente-Manzanares, C. Martinez, and F. Sanchez-Madrid. (1998) The two poles of the lymphocyte: specialized cell compartments for migration and recruitment. Cell Adhes. Commun. 6:125–133.

Dustin, M.L., O. Carpen, and T.A. Springer. (1992) Regulation of locomotion and cell-cell contact area by the LFA-1 and ICAM-1 adhesion receptors. J. Immunol. 148:2654–2663.

Dustin, M. L., S. K. Bromley, Z. Kan, D. A. Peterson, and E. R. Unanue. (1997) Antigen receptor egagement delivers a stop signal to migrating T lymphocytes. Proc. Natl. Acad. Sci. USA 94: 3909–3913.

Dustin, M.L. (1997a) Adhesive bond dynamics in contacts between T lymphocytes and glass-supported planar bilayers reconstituted with the immunoglobulin-related adhesion molecule CD58. J. Biol. Chem. 272:15782–15788.

Elson, E.L., S.F. Felder, P.Y. Jay, M.S. Kolodney, and C. Pasternak. (1999) Forces in cell locomotion. Biochem. Soc. Symp. 65:299–314.

Entschladen, F., B. Niggemann, K.S. Zänker, and P. Friedl. (1997) Differential requirement of protein tyrosine kinases and protein kinase C in the regulation of T cell locomotion in three-dimensional collagen matrices. J. Immunol. 159:3203–3210.

Faull, R.J., N.L. Kovach, J.M. Harlan, and M.H. Ginsberg. (1994) Stimulation of integrin-mediated adhesion of T lymphocytes and monocytes: two mechanisms with divergent biological consequences. J. Exp. Med. 179:1307–1316.

Felder, S. and E.L. Elson. (1990) Mechanics of fibroblast locomotion: quantitative analysis of forces and motions at the leading lamellas of fibroblasts. J. Cell Biol. 111:2513–2526.

Franitzka, S., A. Ronen, and O. Lider. (1999) Real-time analysis of intergrin-mediated chemotactic migration of T lymphocytes within 3-D extracellular matrix-like gels. J. Immunol. Meth. 225:9–25.

Friedl, P., P. B. Noble, and K.S. Zänker. (1993) Lymphocyte locomotion in three-dimensional collagen gels: comparison of three quantitative methods for analysing cell trajectories. J. Immunol. Meth. 165:157–165.

Friedl, P., P.B. Noble, E.D. Shields, and K.S. Zänker. (1994) Locomotor phenotypes of unstimulated CD45RA$^{high}$ and CD45RO$^{high}$ CD4$^{+}$ and CD8$^{+}$ lymphocytes in three-dimensional collagen lattices. Immunology 82:617–624.

Friedl, P., P.B. Noble, and K.S. Zänker. (1995) T Lymphocyte locomotion in a three-dimensional collagen matrix. Expression and function of cell adhesion molecules. J. Immunol. 154:4973–4985.

Friedl, P., K. Maaser, C.E. Klein, B. Niggemann, G. Krohne, and K.S. Zänker. (1997) Migration of highly aggressive MV3 melanoma cells in 3-dimensional collagen lattices results in local matrix reorganization and shedding of alpha2 and beta1 integrins and CD44. Cancer Res. 57:2061–2070.

Friedl, P. and E.-B. Bröcker. (1997) Cancer cell interactions with the extracellular matrix involved in tissue invasion: motility mechanisms beyond the single cell paradigm. In: Heine, H. and Rimpler, M., Extracellular matrix and groundregulation system in health and disease. Stuttgart, Jena, Gustav Fischer Publ., p. 7–18.

Friedl, P., F. Entschladen, C. Conrad, B. Niggemann, and K.S. Zänker. (1998) CD4$^{+}$ T lymphocytes migrating in three-dimensional collagen lattices lack focal adhesions and utilize beta1 integrin-independent strategies for polarization, interaction with collagen fibers and locomotion. Eur. J. Immunol. 28:2331–2343.

Friedl, P., E.-B. Bröcker, and K.S. Zänker. (1998a) Integrins, cell matrix interactions and cell migration strategies: fundamental differences in leukocytes and tumor cells. Cell Adhes.Commun. 6:225–236.

Friedl, P., K.S. Zänker, and E.-B. Bröcker. (1998b) Cell migration strategies in 3-D extracellular matrix: differences in morphology, cell matrix interactions, and integrin function. Microsc. Res. Tech. 43:369–378.

Friedl, P. and E.-B. Bröcker. (2000) The biology of cell locomotion within three-dimensional extracellular matrix. Cell. Mol. Life Sci. 57:41-64.

Friedl, P. and E.-B. Bröcker. (2000a) Biological confocal reflection microscopy: reconstruction of three-dimensional extracellular matrix, cell migration, and matrix reorganization. In: Häder, D.-P., Image analysis in biology. Second edition. CRC Press, Boca Raton, Florida (in press).

Gauthier, D., M. D. Levine, and P. B. Noble. (1991) Principles in object detection for an automated cell tracking system. In: Häder, D.-P. (ed): Image analysis in biology. CRC Press, Boca Raton, Florida, pp 9–28.

Gilat, D., R. Hershkoviz, Y.A. Mekori, I. Vlodavsky, and O. Lider. (1994) Regulation of adhesion of CD4+ T lymphocytes to intact or heparinase- treated subendothelial extracellular matrix by diffusible or anchored RANTES and MIP-1 beta. J. Immunol. 153:4899–4906.

Gimond, C., C. Baudoin, R. van der Neut, D. Kramer, J. Calafat, and A. Sonnenberg. (1998) Cre-loxP-mediated inactivation of the alpha6A integrin splice variant in vivo: evidence for a specific functional role of alpha6A in lymphocyte migration but not in heart development. J. Cell Biol. 143:253–266.

Goebeler, M., D. Kaufmann, E.-B. Bröcker, and C.E. Klein (1996) Migration of highly aggressive melanoma cells on hyaluronic acid is associated with functional changes, increased turnover and shedding of CD44 receptors. J. Cell Sci. 109:1957–1964.

Gretz, J.E., E.P. Kaldjian, A.O. Anderson, and S. Shaw. (1996) Sophisticated strategies for information encounter in the lymph node: the reticular network as a conduit of soluble information and a highway for cell traffic. J. Immunol. 157:495–499.

Gretz, J.E., A.O. Anderson, and S. Shaw. (1997) Cords, channels, corridors and conduits: critical architectural elements facilitating cell interactions in the lymph node cortex. Immunol. Rev. 156:11–24:11–24.

Guido, S. and R.T. Tranquillo. (1993) A methodology for the systematic and quantitative study of cell contact guidance in oriented collagen gels. Correlation of fibroblast orientation and gel birefringence. J. Cell Sci. 105:317–331.

Guilford, W.H., R.C. Lantz, and R.W. Gore. (1995) Locomotive forces produced by single leukocytes in vivo and in vitro. Am. J. Physiol. 268:C1308-C1312.

Gunzer, M., E. Kämpgen, E.-B. Bröcker, K.S. Zänker, and P. Friedl. (1997) Migration of dendritic cells in 3D-collagen lattices: visualisation of dynamic interactions with the substratum and the distribution of surface structures via a novel confocal reflection imaging technique. Adv. Exp. Med. Biol. 417:97–103.

Gunzer, M., A. Schäfer, S. Borgmann, S. Grabbe, K.S. Zänker, E.-B. Bröcker, E. Kämpgen, and P. Friedl. 2000. Antigen presentation in three-dimensional extracellular matrix: productive interactions of T cells with dendritic cells are highly dynamic, short lived, and repetitive.

Haemmerli, G., B. Arnold, and P. Strauli. (1983) Cellular motility on glass and in tissues: similarities and dissimilarities. Cell Biol. Int. Rep. 7:709–725.

Haston, W.S., J.M. Shields, and P.C. Wilkinson. (1982) Lymphocyte locomotion and attachment on two-dimensional surfaces and in three-dimensional matrices. J. Cell Biol. 92:747–752.

Hauzenberger, D., J. Klominek, and K.G. Sundqvist. (1994) Functional specialization of fibronectin-binding beta 1-integrins in T lymphocyte migration. J. Immunol. 153:960–971.

Hauzenberger, D., J. Klominek, S.E. Bergstrom, and K.G. Sundqvist. (1995) T lymphocyte migration: the influence of interactions via adhesion molecules, the T cell receptor, and cytokines. Crit. Rev. Immunol. 15:285–316.

Hauzenberger, D., J. Klominek, J. Holgersson, S.E. Bergstrom, and K.G. Sundqvist. (1997) Triggering of motile behavior in T lymphocytes via cross-linking of alpha 4 beta 1 and alpha L beta 2. J. Immunol. 158:76–84.

Hemler, M.E. (1990) VLA proteins in the integrin family: structures, functions, and their role on leukocytes. Annu.Rev.Immunol. 8:365–400.

Ilic, D., Y. Furuta, S. Kanazawa, K. Takeda, N. Sobue, N. Nakatsuji, S. Nomura, J. Fujimoto, M. Okada, T. Yamamoto, and S. Aizawa. (1995) Reduced cell motility and anhanced focal adhesion contact formation in cells from FAK-deficient mice. Nature 377:539–544.

Islam, L.N. and P.C. Wilkinson. (1992) Restoration with phorbol ester of a locomotor defect in human leukaemic lymphocytes: a visual study of chronic lymphocytic leukaemia cells. Invasion Metastasis 12:47–56.

Jääskeläinen, J., A. Maenpaa, M. Patarroyo, C.G. Gahmberg, K. Somersalo, J. Tarkkanen, M. Kallio, and T. Timonen. (1992) Migration of recombinant IL-2-activated T and natural killer cells in the intercellular space of human H-2 glioma spheroids in vitro. A study on adhesion molecules involved. J. Immunol. 149:260–268.

Kivens, W.J., S.W. Hunt, J.L. Mobley, T. Zell, C.L. Dell, B.E. Bierer, and Y. Shimizu. (1998) Identification of a proline-rich sequence in the CD2 cytoplasmic domain critical for regulation of integrin-mediated adhesion and activation of phosphoinositide 3-kinase. Mol. Cell Biol. 18:5291–5307.

Lauffenburger, D.A. and A.F. Horwitz. (1996) Cell migration: a physically integrated molecular process. Cell 84:359–369.

Leppert, D., Hauser, S. L., Kishiyama, J. L., An, S., Zeng, L., and Goetzl, E. J. (1995) Stimulation of matrix-metalloproteinase-dependent migration of T cells by eicosanoids. FASEB J. 1473–1481.

Lloyd, A.R., J.J. Oppenheim, D.J. Kelvin, and D.D. Taub. (1996) Chemokines regulate T cell adherence to recombinant adhesion molecules and extracellular matrix proteins. J. Immunol. 156:932–938.

Maaser, K., K. Wolf, C. E. Klein, B. Niggemann, K.S. Zänker, E.-B. Bröcker, and P. Friedl. (1999) Functional hierarchy of simultaneously expressed adhesion receptors: Integrin $\alpha 2\beta 1$ but not CD44 mediates MV3 melanoma cell migration and matrix reorganization within three-dimensional hyaluronan-containing collagen matrices. Mol. Biol. Cell. 10:3067-3079.

Maheshwari, G. and Lauffenburger, D.A. (1998) Deconstructing (and reconstructing) cell migration. Microsc. Res. Tech. 43:358–368.

Mandeville, J.T., M.A. Lawson, and F.R. Maxfield. (1997) Dynamic imaging of neutrophil migration in three dimensions: mechanical interactions between cells and matrix. J. Leukoc. Biol. 61:188–200.

Masuyama, J., J.S. Berman, W.W. Cruikshank, C. Morimoto, and D.M. Center. (1992) Evidence for recent as well as long term activation of T cells migrating through endothelial cell monolayers in vitro. J. Immunol. 148:1367–1374.

Mitchison, T.J. and L. P. Cramer. (1996) Actin-based cell motility and cell locomotion. Cell 84: 371–379.

Miyamoto, S., Teramoto, H, Coso, O. A., Gutkind, J. S, Burbelo, P. D., Akiyama, S. K., and Yamada, M. (1999) Integrin function: molecular hierarchies of cytoskeletal and signaling molecules. J.Cell Biol. 131:791–805.

Monks, C.R., B.A. Freiberg, H. Kupfer, N. Sciaky, and A. Kupfer. (1998) Three-dimensional segregation of supramolecular activation clusters in T cells. Nature 395:82–86.

Myat, M.M., S. Andersen, L.A. Allen, and A. Aderem. (1997) MARCKS regulates membrane ruffling and cell spreading. Curr. Biol. 8:611–614.

Negulescu, P.A., T.B. Krasieva, A. Khan, H. Kerschbaum, and M.D. Cahalan. (1996) Polarity of T cell shape, motility and sensitivity to antigen. Immunity 4:421–430.

Nieto, M., M.A. del Pozo, and F. Sanchez-Madrid. (1996) Interleukin-15 induces adhesion receptor redistribution in T lymphocytes. Eur. J. Immunol. 26:1302–1307.

Nieto, M., J.M.R. Frade, D. Sancho, M. Mellado, C. Martinez-A, and F. Sanchez Madrid. (1997) Polarizing of chemokine receptors to the leading edge during lymphocyte chemotaxis. J. Exp. Med. 186:153–158.

Niewöhner, J., Weber, I., Maniak, M., Müller-Tauchenberger, A., and Gerisch, G. (1997) Talin-null cells from Dictyostelium are strongly defective in adhesion to particle and substrate surfaces and slightly impaired in cytokinesis. J. Cell Biol. 138:349–361.

Nikolai, G., B. Niggemann, M. Werner, K.S. Zänker, and P. Friedl. (1998) Direct and rapid induction of migration in human $CD4^+$ T lymphocytes within three-dimensional collagen matrices mediated by signalling via CD3 and/or CD2. Immunology 95:62–68.

Nikolai, G., Niggemann, B., Werner, M., and Zänker, K. S. (1999) Colcemid but not taxol modulates the migratory behavior of human T lymphocytes within 3-D collagen lattices. Immunobiol. 201:107-119.

Ohtani, O., A. Kikuta, A. Ohtsuka, and T. Murakami. (1991) Organization of the reticular network of rabbit Peyer's patches. Anat. Rec. 229:251–258.

Ohtsuka, A., A.J. Piazza, T.H. Ermak, and R.L. Owen. (1992) Correlation of extracellular matrix components with the cytoarchitecture of mouse Peyer's patches. Cell Tissue Res. 269:403–410.

Palecek, S.P., J.C. Loftus, M.H. Ginsberg, D.A. Lauffenburger, and A.F. Horwitz. (1997) Integrin-ligand binding properties govern cell migration speed through cell-substratum adhesiveness. Nature 385:537–540.

Ratner, S. (1992) Lymphocyte migration through extracellular matrix. Invasion Metastasis 12:82–100.

Ratner, S., W.S. Sherrod, and D. Lichlyter. (1997) Microtubule retraction into the uropod and its role in T cell polarization and motility. J. Immunol. 159:1063–1067.

Reid, G.G. and I. Newman. (1991) Human leucocyte migration through collagen matrices containing other extracellular matrix components. Cell Biol. Int. Rep. 15:711–720.

Romzek, N.C., E.S. Harris, C.L. Dell, J. Skronek, E. Hasse, P.J. Reynolds, S.W. Hunt, and Y. Shimizu. (1998) Use of a beta1 integrin-deficient human T cell to identify beta1 integrin cytoplasmic domain sequences critical for integrin function. Mol. Biol. Cell 9:2715–2727.

Saltzman, W.M., M.R. Parkhurst, P. Parsons-Wingerter, and W.H. Zhu. (1992) Three-dimensional cell cultures mimic tissues. Ann. N.Y. Acad. Sci. 665:259–73:259–273.

Sanchez-Madrid, F. and M.A. del Pozo. (1999). Leukocyte polarization in cell migration and immune interactions. EMBO J. 18:501-511.

Sanchez-Mateos, P., M.R. Campanero, M.A. del Pozo, and F. Sanchez-Madrid. (1995) Regulatory role of CD43 leukosialin on integrin-mediated T-cell adhesion to endothelial and extracellular matrix ligands and its polar redistribution to a cellular uropod. Blood 86:2228–2239.

Schor, S.L., T.D. Allen, and B. Winn. (1983) Lymphocyte migration into three-dimensional collagen matrices: a quantitative study. J. Cell Biol. 96:1089–1096.

Serrador, J.M., J.L. Alonso-Lebrero, M.A. del Pozo, H. Furthmayr, R. Schwartz-Albiez, J. Calvo, F. Lozano, and F. Sanchez-Madrid. (1997) Moesin interacts with the cytoplasmic region of intercellular adhesion molecule-3 and is redistributed to the uropod of T lymphocytes during cell polarization. J. Cell Biol. 138:1409–1423.

Shaw, A.R., A. Domanska, A. Mak, A. Gilchrist, K. Dobler, L. Visser, S. Poppema, L. Fliegel, M. Letarte, and B.J. Willett. (1995) Ectopic expression of human and feline CD9 in a human B cell line confers beta 1 integrin-dependent motility on fibronectin and laminin substrates and enhanced tyrosine phosphorylation. J. Biol. Chem. 270:24092–24099.

Shaw, A.S. and M.L. Dustin. (1997) Making the T cell receptor go the distance: a topological view of T cell activation. Immunity 6:361–369.

Sheetz, M.P., D.P. Felsenfeld, and C.G. Galbraith. (1998) Cell migration: regulation of force on extracellular-matrix-integrin complexes. Trends Cell Biol. 8:51–54.

Shields, J.M., W. Haston, and P.C. Wilkinson. (1984) Invasion of collagen gels by mouse lymphoid cells. Immunology 51:259–268.

Shimizu, Y. and S. Shaw. (1991) Lymphocyte interactions with extracellular matrix. FASEB J. 5:2292–2299.

Springer, T.A. (1994) Traffic signals for lymphocyte recirculation and leukocyte emigration: the multistep paradigm. Cell 76:301–314.

Stössel, T.P. (1994) The E. Donnall Thomas Lecture (1993): The machinery of blood cell movements. Blood 84:367–379.

Sundqvist, K.G., D. Hauzenberger, K. Hultenby, and S.E. Bergström. (1993) T lymphocyte infiltration of two- and three-dimensional collagen substrata by an adhesive mechanism. Exp. Cell Res. 206:100–110.

Sundqvist, K.G., L. Pedari, and D. Hauzenberger. (1993a) Anchorage and lymphocyte function: extracellular matrix substrata control morphogenesis and interleukin production but have minor effects on DNA synthesis. Scand. J. Immunol. 37:295–307.

Taub, D.D., G. Tsarfaty, A.R. Lloyd, S.K. Durum, D.L. Longo, and W.J. Murphy. (1994) Growth hormone promotes human T cell adhesion and migration to both human and murine matrix proteins in vitro and directly promotes xenogeneic engraftment. J.Clin.Invest. 94:293–300.

Taylor, D.L. and J.S. Condeelis. (1979) Cytoplasmic structure and contractility in amoeboid cells. Int. Rev. Cytol. 56:57–144:57–144.

Taylor, D.L., P.L. Moore, J.S. Condeelis, and R.D. Allen. (1976). The mechanochemical basis of amoeboid movement. I. Ionic requirements for maintaining viscoelasticity and contractility of Amoeba cytoplasm. Exp. Cell Res. 101:127–133.

Tsukita, S. and S. Yonemura. (1997) ERM (ezrin/radixin/moesin) family: from scytoskeleton to signal transduction. Curr. Opin. Cell Biol. 9:70–75.

Turley, E.A., C.A. Erickson, and R.P. Tucker. (1985. The retention and ultrastructural appearances of various extracellular matrix molecules incorporated into three-dimensional hydrated collagen lattices. Dev. Biol. 109:347–369.

Vicente-Manzanares, M., M.C. Montoya, M. Mellado, J.M. Frade, M.A. del Pozo, M. Nieto, M.O. de Landazuri, A. Martinez, and F. Sanchez-Madrid. (1998) The chemokine SDF-1α triggers a chemotactic response and induces cell polarization in human B lymphocytes. Eur. J. Immunol. 28:2197–2207.

Werr, J., X. Xie, P. Hedqvist, E. Ruoslahti, and L. Lindbom. (1998) beta1 integrins are critically involved in neutrophil locomotion in extravascular tissue In vivo. J. Exp. Med. 187:2091–2096.

Wilkinson, P.C., J.M. Shields, and W.S. Haston. (1983) Contact guidance of locomotion of human leukocytes. Agents Actions Suppl. 12:91–105:91–105.

Wilkinson, P.C. (1986) The locomotor capacity of human lymphocytes and its enhancement by cell growth. Immunology 57:281–289.

Wilkinson, P.C. and I. Newman. (1992) Identification of IL-8 as a locomotor attractant for activated human lymphocytes in mononuclear cell cultures with anti-CD3 or purified protein derivative of Mycobacterium tuberculosis. J. Immunol. 149:2689–2694.

Woodside, D.G., D.K. Wooten, and B.W. McIntyre. (1998) Adenosine diphosphate (ADP)-ribosylation of the guanosine triphosphatase (GTPase) rho in resting peripheral blood human T lymphocytes results in pseudopodial extension and the inhibition of T cell activation. J. Exp. Med. 188:1211–1221.

Zell, T., C.S. Warden, A.S. Chan, M.E. Cook, C.L. Dell, S.W. Hunt, and Y. Shimizu. (1998) Regulation of beta 1-integrin-mediated cell adhesion by the Cbl adaptor protein. Curr. Biol. 8:814–822.

